# Cellular and Molecular Mechanisms Regulating Neuronal Function, Homeostasis, and Disease

**DOI:** 10.3390/brainsci16050513

**Published:** 2026-05-12

**Authors:** Ana Filipa Sobral, Daniel José Barbosa

**Affiliations:** 1 Associate Laboratory i4HB—Institute for Health and Bioeconomy, University Institute of Health Sciences—CESPU, 4585-116 Gandra, Portugal; 2 UCIBIO—Applied Molecular Biosciences Unit, Toxicologic Pathology Research Laboratory, University Institute of Health Sciences (1H-TOXRUN, IUCS-CESPU), 4585-116 Gandra, Portugal; 3 UCIBIO—Applied Molecular Biosciences Unit, Translational Toxicology Research Laboratory, University Institute of Health Sciences (1H-TOXRUN, IUCS-CESPU), 4585-116 Gandra, Portugal

## 1. Introduction

The brain is an extremely complex organ whose function depends on the precise coordination of molecular, cellular, and network processes. The homeostatic balance of the brain is maintained through tightly regulated interactions involving intracellular signaling pathways, mitochondrial activity, endoplasmic reticulum function, gene expression programs, communication between glial cells and neurons, and neurovascular coupling [1]. Disruption of these interconnected systems is increasingly recognized as a central feature of a wide range of neurological and neurodevelopmental disorders, including neurodegenerative diseases, brain injury, and developmental disorders [2,3].

Recent advances in neuroscience have significantly expanded our understanding of the cellular mechanisms underlying brain function. In particular, growing evidence highlights the importance of organelle crosstalk, such as mitochondria-associated endoplasmic reticulum signaling, in influencing neuronal vulnerability under metabolic stress conditions [4]. At the same time, transcriptomic and biological approaches have revealed that brain aging exerts a profound and dominant influence on gene expression patterns, frequently modulating or even surpassing the effects of genetic risk factors [5]. In parallel, developmental neuroscience has increasingly revealed complex gene regulatory networks that guide early brain development [6], while research on brain injury has shifted from focusing only on lesions toward a broader network-based view of structural and molecular changes after damage [7]. Additionally, the roles of astrocytes and the blood–brain barrier (BBB) have gained increased attention, particularly in the context of intracellular transport mechanisms and cytoskeletal dynamics that sustain neurovascular integrity [8].

Despite these advances, important gaps in knowledge persist. The integration of molecular mechanisms across different biological levels, ranging from subcellular organelles to whole-brain systems, is still incomplete [9]. Furthermore, the relative contribution of aging, genetics, and environmental or metabolic stress to brain dysfunction is still not fully understood, particularly in clinical contexts [3]. Similarly, the mechanisms behind network responses to focal brain injury are not yet fully characterized [10], and the functional relevance of many developmental gene networks remains unclear [11]. All these gaps limit the ability to design targeted and effective therapeutic strategies for neurological disorders.

This Special Issue aimed to address these challenges by bringing together six original research articles that explore complementary aspects of brain function and dysfunction. Collectively, these studies explored the role of astrocytes in regulating BBB function (contribution 1), developmental gene expression networks in the cerebellum during perinatal development (contribution 2), responses of contralateral cerebral hemisphere to brain injury (contribution 3), transcriptomic determinants of brain aging (contribution 4), mitochondrial and endoplasmic reticulum interactions affecting neuronal communication in diabetes (contribution 5), and lipid-mediated modulation of neuronal bioenergetics (contribution 6). By spanning multiple levels of analysis and diverse biological contexts, this Special Issue provides an integrated perspective on the mechanisms that shape brain homeostasis and its disruption.

Importantly, this Special Issue also highlights emerging directions for future research, including the need for multiscale integration of molecular, cellular, and system data; deeper characterization of aging as a dominant biological driver; improved understanding of brain-wide responses to localized injury; and further elucidation of glial and neurovascular mechanisms. These directions are expected to stimulate new conceptual and methodological advances in the field.

The aim of this Editorial is therefore to briefly contextualize the published contributions, highlight their key findings, and emphasize their collective relevance to advancing our understanding of brain function in health and disease.

## 2. An Overview of Published Articles

Sobral, A. F. et al. (contribution 1) addressed the role of molecular motors in BBB maintenance by astrocytes. Astrocytes are essential regulators of the neurovascular unit, contributing to barrier integrity, nutrient exchange, and homeostatic signaling. The authors highlighted how intracellular transport systems and cytoskeletal motor proteins participate in astrocytic support of the BBB. By reviewing research that links molecular trafficking mechanisms to vascular homeostasis, this work expanded our understanding of how astrocyte dysfunction may contribute to neurological diseases.

Vaganova, A. N. et al. (contribution 2) investigated *Taar5* expression dynamics and its associated co-expression networks in the cerebellum during perinatal development using transcriptomic profiling approaches. The authors characterized developmental stage-dependent expression patterns of the *Taar5* gene and identified gene modules co-expressed with this receptor that are enriched in processes related to cerebellar development. Their network-based analysis suggests that Taar5 may participate in broader regulatory programs active during critical periods of cerebellar development, supporting a potential role for Taar5 receptor signaling in influencing early cerebellar maturation. Given the expanding understanding of cerebellar involvement in cognitive and affective functions beyond motor control, these findings provide a molecular basis for exploring how neuromodulatory receptor systems contribute to cerebellar development and their potential relevance to neurodevelopmental diseases.

Liao, X. et al. (contribution 3) explored the contralateral effects of severe unilateral brain injury. Although brain lesions are commonly studied at the primary injury site, increasing evidence indicates that contralesional regions also undergo substantial changes [12]. In this study, the authors identified molecular responses (increased levels of synapsin-1 and postsynaptic density protein 95—PSD95) and structural changes (destruction of dendritic structures and the encapsulation of astrocytes by synapses) in the motor cortex contralateral to the lesion. Moreover, genome-wide transcriptome analysis revealed a marked upregulation of pathways related to inflammation, synaptic function, and axonal regeneration in the contralateral cortex following injury, suggesting that severe unilateral injury triggers contralateral adaptations. These results emphasize the existence of interconnected networks that modulate brain diseases and suggest that therapeutic strategies should consider whole-brain responses rather than focusing only on injured areas.

Labuza, A. et al. (contribution 4) focused on one of the most discussed genetic risk determinant for late-onset Alzheimer’s disease (AD), the apolipoprotein E (APOE) genotype [13]. Using mouse models expressing human APOE2, APOE3, and APOE4, the authors analyzed brain transcriptomic changes to determine the relative influence of genotype and aging. Their results showed that while the APOE genotype does influence specific molecular pathways, the overall gene expression profile of the brain is more strongly influenced by aging. This study suggests that chronological aging may have a greater impact than the APOE genotype in determining brain molecular signatures. These findings help refine the current understanding of genetic risk factors in neurodegeneration and highlight the need to consider aging as a central factor when interpreting APOE-related effects in AD.

Zhang, J. et al. (contribution 5) investigated the mechanisms underlying synaptic dysfunction in diabetic mice, focusing on mitochondria-associated endoplasmic reticulum membranes (MAMs). These structures are critical hubs for communication between the endoplasmic reticulum and mitochondria, particularly in Ca^2+^ transfer and metabolic regulation [4]. The authors demonstrated that diabetes increases MAM activity, leading to excessive mitochondrial Ca^2+^ uptake, which was associated with mitochondrial dysfunction and promoted endoplasmic reticulum stress. Importantly, these cellular perturbations were closely linked to impaired synaptic plasticity in the brain, suggesting a direct functional consequence at the level of neuronal communication. The study further supports the idea that disrupted MAM signaling acts as a critical mechanism connecting systemic metabolic disease to neuronal stress responses. Overall, the findings support the concept that altered organelle crosstalk may play a central role in diabetic brain complications, including cognitive decline.

Lastly, Jeon, S.-M. et al. (contribution 6) examined how the purity of phosphatidylserine influences the function of human cortical neurons. Phosphatidylserine is a membrane phospholipid with recognized importance in neuronal physiology [14], but the significance of compound purity on its therapeutic potential remains underexplored. The study showed that higher-purity phosphatidylserine produced stronger improvements in neuronal performance by modulating the sirtuin 1 (SIRT1)-peroxisome proliferator-activated receptor gamma coactivator 1-alpha (PGC-1α) pathway, a key signaling axis involved in mitochondrial biogenesis, energy metabolism, and cellular resilience. Moreover, in an amyloid-beta 1–42 (Aβ_42_)-induced AD model, higher-purity phosphatidylserine also showed better protective effects on neuronal viability and signaling. Overall, the study highlights that not only the presence of phosphatidylserine but also its purity can significantly influence its biological effects in neurons, particularly through mitochondrial-related pathways.

## 3. Conclusions

The contributions collected in this Special Issue collectively advance the understanding of the molecular and cellular mechanisms that regulate brain function across development, aging, metabolic diseases, and injury. Together, they emphasize that brain homeostasis emerges from the integration of multiple interacting systems rather than isolated pathways.

A repeated theme across studies is the central role of intracellular and intercellular communication. Developmental and injury-related studies demonstrated that brain function is dynamically regulated across time and space, with gene expression programs and structural responses extending beyond localized regions. In parallel, transcriptomic analyses highlighted aging as a dominant force shaping brain molecular landscapes, often surpassing the influence of genetic background. Additionally, mitochondrial dysfunction, endoplasmic reticulum stress, and altered lipid-mediated signaling emerged as key drivers of neuronal vulnerability, particularly in metabolic and bioenergetic contexts.

Methodologically, the studies included in this Special Issue illustrate the value of combining animal models, human neuronal systems, transcriptomic profiling, and cellular and molecular analyses. This diversity of approaches enables a more comprehensive understanding of brain physiology and supports the identification of cooperative mechanisms across different experimental contexts.

These studies also reinforce the need to move toward integrative and multiscale approaches. Understanding brain function and dysfunction requires connecting molecular mechanisms to cellular behavior and brain network organization. This is particularly relevant to translational neuroscience, where therapeutic strategies need to consider the complex and multifactorial nature of brain disorders.

In conclusion, this Special Issue provides a clear understanding of how molecular and cellular processes influence brain function in both health and disease. The findings presented here expand current knowledge and highlight important future directions, including improved integration of multi-omics data, a clearer understanding of aging-related mechanisms, and further investigation of the roles of glial cells and neurovascular systems in brain resilience and dysfunction. We hope that this collection will stimulate continued research and foster new conceptual advances in neuroscience.

## Abbreviations

The following abbreviations are used in this manuscript:

Aβ_42_	Amyloid-beta 1–42
AD	Alzheimer’s disease
APOE	Apolipoprotein E
BBB	Blood-brain barrier
MAMs	Mitochondria-associated endoplasmic reticulum membranes
PGC-1α	Peroxisome proliferator-activated receptor gamma coactivator 1-alpha
PSD95	Postsynaptic density protein 95
SIRT1	Sirtuin 1
